# Contrast Enhancement of the Olfactory Recess Using Heavily T2-Weighted 3D Fluid-Attenuated Inversion Recovery Imaging (FLAIR)

**DOI:** 10.7759/cureus.82153

**Published:** 2025-04-12

**Authors:** Koichiro Matsuura, Iichiro Osawa, Keita Nagawa, Shinji Kakemoto, Kaiji Inoue, Eito Kozawa

**Affiliations:** 1 Radiology, Saitama Medical University Hospital, Saitama, JPN

**Keywords:** 3d flair, contrast enhancement, cribriform plate, olfactory nerve, olfactory recess

## Abstract

Purpose

This study aimed to assess the contrast enhancement of the olfactory recess using heavily T2-weighted 3D fluid-attenuated inversion recovery (FLAIR) imaging after intravenous gadolinium administration.

Methods

This retrospective study included 35 patients. The overall contrast enhancement of the bilateral olfactory recess between the precontrast, postcontrast, and 4-h delayed postcontrast T2-weighted 3D FLAIR images were subjectively evaluated. In addition, serial changes in signal intensity in the olfactory recess and other structures (other cerebrospinal fluid spaces and the pons) were objectively assessed.

Results

Subjective analysis showed that contrast enhancement of the olfactory recess was observed in 61 (right: 28, left: 33) and 67 (right: 32, left: 35) of 70 ears from 35 patients on postcontrast and 4-h delayed postcontrast T2-weighted 3D FLAIR images, respectively. In the objective analysis, the relative enhancement of the olfactory recess was the highest for 4-h delayed postcontrast images, followed by postcontrast images. This enhancement pattern was similar to that of the cerebrospinal fluid spaces surrounding the other cranial nerves.

Conclusion

The olfactory recess was enhanced using T2-weighted 3D FLAIR imaging. These findings may provide novel insights into cerebrospinal fluid and solute dynamics and may contribute to optimizing imaging protocols for evaluating related neurological disorders.

## Introduction

 The olfactory recess (or groove) is a depression in the anterior cranial cavity. Its floor is composed of the cribriform plate of the ethmoid bone. The olfactory bulb, surrounded by the cerebrospinal fluid (CSF), lies within the olfactory recess. The cribriform plate is a sieve-like structure with numerous perforations through which first-order olfactory nerves ascend intracranially and synapse with second-order neurons within the olfactory bulb. Nasal lymphatic outflow via the cribriform plate is a major route of CSF efflux to the nasal mucosa in animals [1]. However, this pathway has not been fully investigated in humans [1].

An evaluation of contrast enhancement in intracranial structures is essential for a detailed understanding of CSF and solute dynamics. Heavily T2-weighted (T2W) 3D fluid-attenuated inversion recovery (FLAIR) (HT2-FLAIR) imaging is a type of 3D FLAIR imaging that provides greater sensitivity to subtle T1-shortening caused by, for example, low concentrations of gadolinium or increased protein levels compared to conventional 3D FLAIR imaging [2-5]. This technique was also used to evaluate subtle T1-shortening effects in various regions such as CSF spaces [2,3], the inner ear [4], and CSF leaks [5,6]. Since nasal lymphatic outflow is a potential CSF drainage pathway, evaluating contrast enhancement of the olfactory recess could offer valuable information regarding CSF dynamics as well as related disorders.

However, to the best of our knowledge, contrast enhancement of the olfactory recess using HT2-FLAIR imaging after intravenous administration of gadolinium has not yet been reported. The purpose of our study was to assess contrast enhancement using this imaging technique. This contrast enhancement may provide insights into CSF-related neurological disorders (e.g., hydrocephalus) or neurodegenerative diseases (e.g., Alzheimer’s disease).

## Materials and methods

Patients

Imaging records of 36 consecutive patients with suspected endolymphatic hydrops were retrospectively reviewed between March 2017 and January 2022. The inclusion criteria were as follows: 1) availability of HT2-FLAIR imaging with and without a gadolinium-based contrast agent and 2) availability of magnetic resonance (MR) cisternography (MRC). The exclusion criteria were as follows: 1) insufficient image quality to interpret the images, 2) the olfactory recess not included in the field of view (FOV), 3) small CSF spaces to place regions of interest (ROIs) on, and 4) observation of intracranial lesions, such as hemorrhage or tumors, directly influencing the CSF spaces. Insufficient image quality to interpret the images was defined as the presence of severe noise and artifacts such as motion and metal artifacts, which interfered with image quality and interpretation.

The Institutional Review Board of Saitama Medical University Hospital, Saitama, Japan, approved the study design. We obtained written informed consent for the procedures and opt-out consent for the use of retrospective clinical data from all patients or the parents/legal guardians of patients aged < 18 years.

MR imaging acquisition

**Figure 1:**
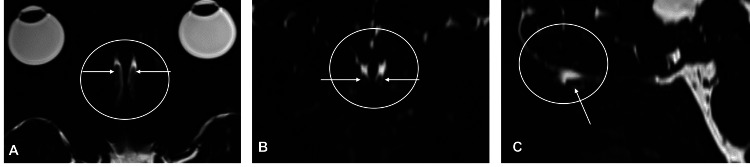
Magnetic resonance cisternography (MRC) of the olfactory recess and olfactory nerve A-C: The olfactory recess (circle) is hyperintense on the axial (A), coronal reformatted (B), and sagittal reformatted (C) MRC. The olfactory nerve (arrow) is located in the olfactory recess.

All patients underwent a 3 Tesla MR examination (MAGNETOM Skyra, Siemens, Erlangen, Germany) with a 32-channel head coil. Thirty-six patients underwent HT2-FLAIR imaging and MRC (Figure 1) of the inner ear using a protocol for endolymphatic hydrops assessment [2]. Table 1 summarizes the detailed parameters of HT2-FLAIR imaging and MRC. Postcontrast HT2-FLAIR images were obtained approximately 0.5 to 5 min after an intravenous injection of gadolinium-HP-DO3A (Gadoteridol) at a single dose of 0.2 mL/kg (0.1 mmol/kg), followed by delayed postcontrast HT2-FLAIR imaging taken at 4 h. Postcontrast imaging time points were chosen based on prior studies [2,3] demonstrating chronological changes in the contrast enhancement of CSF spaces.

**Table 1:** Magnetic resonance (MR) imaging parameters FLAIR: fluid-attenuated inversion recovery, HT2-FLAIR: heavily T2-weighted 3D FLAIR, MRC: MR cisternography, TR: repetition time, TE: echo time, FOV: field of view, GRAPPA: Generalized Autocalibrating Partially Parallel Acquisition

	HT2-FLAIR imaging	MRC
TR/TE (ms)	9000/542	4400/542
Inversion time (ms)	2250	-
Frequency-selective fat suppression prepulse	+	+
Flip angle	Average flip angle 120°	Average flip angle 120°
Echo train length	519	519
Acquisition matrix size	324 × 384	324 × 384
FOV (mm^2^)	166 × 196	166 × 196
Axial slices	104	104
In-plane resolution (mm)	0.5 × 0.5	0.5 × 0.5
Slice thickness (mm)	1.0	1.0
Bandwidth (Hz/pixel)	434	434
Acceleration factor	2 using GRAPPA	2 using GRAPPA
Number of excitations	2	1.8
Total time (min.s)	7.17	3.15

MR imaging analysis

Imaging analysis was performed subjectively and objectively using a workstation (Synapse VINCENT version 5.2; Fuji Film, Tokyo, Japan). All images were reviewed by a board-certified radiologist (K.M.) who was blinded to clinical information.

Subjective analysis

For subjective analysis, the radiologist visually assessed the overall contrast enhancement of the olfactory recess. The presence of contrast enhancement was defined as increased signal intensity (SI) on postcontrast or 4-h delayed postcontrast HT2-FLAIR imaging compared to precontrast imaging. In addition, SI was compared between postcontrast and 4-h delayed postcontrast HT2-FLAIR imaging and divided into three groups: higher postcontrast imaging, equal, and higher 4-h delayed postcontrast imaging. Finally, the difference in contrast enhancement of the olfactory recess between the left and right sides on postcontrast and 4-h delayed postcontrast HT2-FLAIR imaging were determined: difference (one with enhancement and the other without enhancement) or no difference (bilateral enhancement or bilateral no enhancement).

Objective analysis

For objective analysis, the radiologist measured the SI in predefined ROIs. ROIs were predefined based on anatomical landmarks known to be relevant in previous studies on CSF enhancement [2,3] and manually placed within the following structures on HT2-FLAIR imaging with reference to the corresponding MRC: 1) olfactory recess; 2) CSF spaces, including CSF spaces surrounding the cranial nerves (the orbital CSF space, Meckel’s cave, and fundus of the internal auditory canal), olfactory sulcus, lateral ventricle, and prepontine cistern; and 3) brainstem (pons). For the olfactory recess, ovoid ROIs (1 mm^2^) were placed on the left and right sides, and the SI was calculated as the mean of both sides.

For the CSF spaces, ovoid ROIs (1 mm^2^) were drawn on the left and right sides of the CSF spaces surrounding the cranial nerve, olfactory sulcus, and lateral ventricle. The SI was calculated as the mean of both sides. An ovoid ROI (3 mm^2^) was placed at the center of the prepontine cistern. Regarding the brainstem, an ovoid ROI (20 mm^2^) was placed at the center of the pons.

The mean SI was calculated at each time point. SI measurements were performed, and the final data were summarized as means and standard deviations (SD). Finally, the SI at each time point was corrected for relative enhancement by normalizing it to the precontrast SI.

Statistical analysis

Repeated measurements of relative enhancement at three different time points were evaluated using the Friedman test, followed by Holm correction for multiple comparisons. All statistical calculations were conducted using SPSS (version 27.0; IBM, Armonk, NY, USA) and the statistical computing language R (R Foundation for Statistical Computing, Vienna, Austria). P values < 0.05 were considered to be significant.

## Results

This study visually identified distinct contrast enhancement patterns in the olfactory recess using HT2-FLAIR imaging, which were comparable to CSF spaces surrounding the other cranial nerves. Relative enhancement of the olfactory recess increased over time, with statistical significance across all measured time points.

Patients

Thirty-six patients underwent MRC and HT2-FLAIR imaging, with and without gadolinium-based contrast agents. Thirty-five of the 36 patients with available images fulfilled all the inclusion criteria and none of the exclusion criteria. One patient was excluded because the CSF space was compressed owing to bilateral chronic subdural hematomas. Finally, this study included 16 men and 19 women with an average age of 53 years (range, 17-80 years).

Subjective analysis

**Figure 2:**
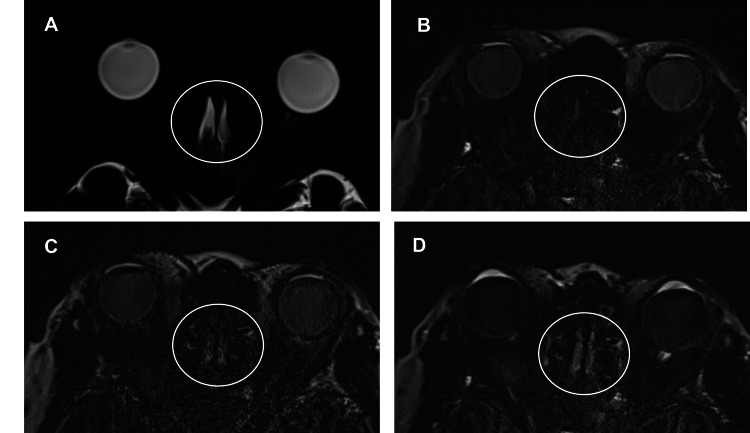
Serial changes in contrast enhancement of the olfactory recess A: The olfactory recess (circle) on magnetic resonance cisternography (MRC). B-D: The olfactory recess (circle) on precontrast (B), postcontrast (C), and 4-h delayed postcontrast (D) HT2-FLAIR imaging. The olfactory recess shows contrast enhancement on postcontrast (C) and 4-h delayed postcontrast (D) heavily T2-weighted (T2W) fluid-attenuated inversion recovery (FLAIR) (HT2-FLAIR) imaging. Contrast enhancement is higher on 4-h delayed postcontrast imaging than on postcontrast imaging.

Table 2 summarizes the results of the visual analysis of contrast enhancement in the olfactory recess. Figure 2 shows the serial changes in contrast enhancement of the olfactory recess. In 70 ears from 35 patients, contrast enhancement was observed in 61 (right: 28, left: 33; 87%) and 67 (right: 32, left: 35; 96%) ears on postcontrast and 4-h delayed postcontrast HT2-FLAIR images, respectively. Tables 3, 4 show the differences in the overall SI of the olfactory recess between postcontrast and 4-h delayed postcontrast imaging and differences in contrast enhancement between the left and right sides, respectively. The overall SI of the olfactory recess was higher on 4-h delayed postcontrast imaging than on postcontrast imaging in all patients. When comparing contrast enhancement between the left and right sides, the “no difference” category was observed more frequently than the “difference” category.

**Table 2:** Contrast enhancement of the olfactory recess through subjective visual analysis FLAIR: fluid-attenuated inversion recovery, HT2-FLAIR: heavily T2-weighted 3D FLAIR, Post: postcontrast HT2-FLAIR imaging, 4-h post: 4-h delayed postcontrast HT2-FLAIR imaging, Rt: right, Lt: left “n” represents the number of patients. Figures in parentheses indicate percentages to the total.

		Contrast enhancement			
		Post		4-h post	
		Rt	Lt	Rt	Lt
Olfactory recess	n (%)	28 (80%)	33 (94%)	32 (91%)	35 (100%)

**Table 3:** Differences in signal intensity (SI) of the olfactory recess through subjective visual analysis FLAIR: fluid-attenuated inversion recovery, HT2-FLAIR: heavily T2-weighted 3D FLAIR, Post: postcontrast HT2-FLAIR imaging, 4-h post: 4-hour delayed postcontrast HT2-FLAIR imaging “n” represents the number of patients. Figures in parentheses indicate percentages to the total.

		Differences in SI between post vs. 4-h post		
		Higher post	Equal	Higher 4-h post
Olfactory recess	n (%)	0 (0%)	0 (0%)	35 (100%)

**Table 4:** Laterality differences in contrast enhancement of the olfactory recess through subjective visual analysis FLAIR: fluid-attenuated inversion recovery, HT2-FLAIR: heavily T2-weighted 3D FLAIR, Post: postcontrast HT2-FLAIR imaging, 4-h post: 4-hour delayed postcontrast HT2-FLAIR imaging “n” represents the number of patients. Figures in parentheses indicate percentages to the total.

		Differences in contrast enhancement between left vs. right sides			
		Post		4-h post	
		Difference	No difference	Difference	No difference
Olfactory recess	n (%)	8 (23%)	27 (77%)	3 (9%)	32 (91%)

Objective analysis

**Figure 3:**
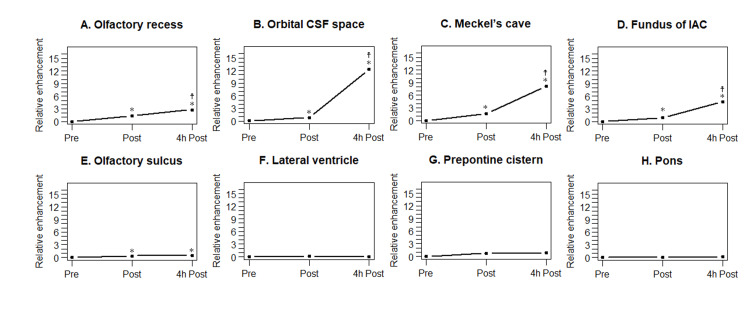
Relative enhancement plots for the olfactory recess and other structures A: Relative enhancement plots for the olfactory recess. Relative enhancement is statistically significantly higher on postcontrast images compared to that on precontrast images, and it is statistically significantly higher on 4-h delayed postcontrast images compared to that on postcontrast images. B-D: Relative enhancement plots for cerebrospinal fluid (CSF) spaces surrounding the cranial nerves (B: orbital CSF space, C: Meckel’s cave, and D: fundus of the internal auditory canal). A contrast enhancement pattern similar to the olfactory recess is observed in these spaces. E: Relative enhancement plots for the olfactory sulcus. Relative enhancement is statistically significantly higher on postcontrast and 4-h delayed postcontrast images compared to that on precontrast images. However, no statistically significant difference in relative enhancement is observed between 4-h delayed postcontrast and postcontrast images. F-H: Relative enhancement plots for the lateral ventricle (F), prepontine cistern (G), and pons (H). In all of these regions, no statistically significant difference is observed in relative enhancement between the three time points. Asterisk (*) and cross of Lorraine (☨) indicate statistical significance (p < 0.05) versus relative enhancement on precontrast and postcontrast imaging, respectively.

Table 5 summarizes the relative enhancements of the olfactory recess and other structures. Figure 3 shows the relative enhancement plots. Table 6 shows the corresponding p values, indicating the statistical significance of the differences in the relative enhancement at three different time points. Relative enhancement in the olfactory recess was statistically significantly higher on 4-h delayed postcontrast HT2-FLAIR imaging than on precontrast and postcontrast imaging. Relative enhancement was also statistically significantly higher on post-contrast imaging than on precontrast imaging. The CSF spaces surrounding the cranial nerves exhibited an enhancement pattern similar to that of the olfactory recess. The olfactory sulcus demonstrated slight contrast enhancement on postcontrast and 4-h delayed postcontrast images. However, no statistically significant differences were observed between the postcontrast and 4-h delayed postcontrast images. In the lateral ventricle, prepontine cistern, and pons, there was no statistically significant difference in the relative enhancement between precontrast, postcontrast, and 4-h delayed postcontrast imaging.

**Table 5:** Relative enhancement of the olfactory recess and other structures FLAIR: fluid-attenuated inversion recovery, HT2-FLAIR: heavily T2-weighted 3D FLAIR, SD: standard deviation, Post: postcontrast HT2-FLAIR imaging, 4-h post: 4-h delayed postcontrast HT2-FLAIR imaging, CSF: cerebrospinal fluid, IAC: internal auditory canal Data are represented as means and SD (in parentheses).

	Relative enhancement (SD)	
	Post	4-h post
Olfactory recess	1.35 (1.37)	2.30 (1.74)
Orbital CSF space	1.42 (1.85)	11.54 (8.39)
Meckel’s cave	0.58 (0.65)	6.24 (5.82)
Fundus of IAC	0.97 (0.80)	10.26 (7.57)
Olfactory sulcus	0.31 (0.56)	0.52 (0.81)
Lateral ventricle	0.17 (0.71)	0.06 (1.75)
Prepontine cistern	0.11 (0.73)	0.28 (0.85)
Pons	0.03 (0.14)	0.06 (0.18)

**Table 6:** Comparison of relative enhancement and statistical significance (p values) across three time points FLAIR: fluid-attenuated inversion recovery, HT2-FLAIR: heavily T2-weighted 3D FLAIR, Post: postcontrast HT2-FLAIR imaging, 4-h post: 4-h delayed postcontrast HT2-FLAIR imaging, CSF: cerebrospinal fluid, IAC: internal auditory canal Asterisks (*) indicate statistically significant differences (p < 0.05).

	p value		
	Pre vs. Post	Pre vs. 4-h post	Post vs. 4-h post
Olfactory recess	< 0.001*	< 0.001*	< 0.001*
Orbital CSF space	< 0.001*	< 0.001*	< 0.001*
Meckel’s cave	< 0.001*	< 0.001*	< 0.001*
Fundus of IAC	< 0.001*	< 0.001*	< 0.001*
Olfactory sulcus	0.006*	0.004*	0.266
Lateral ventricle	0.188	0.838	0.611
Prepontine cistern	0.395	0.061	0.099
Pons	0.186	0.054	0.475

## Discussion

To the best of our knowledge, the present study is the first to demonstrate contrast enhancement of the olfactory recess on HT2-FLAIR imaging following intravenous gadolinium administration, with stronger enhancement observed on 4-h delayed postcontrast imaging than on postcontrast imaging. The enhancement pattern of the olfactory recess was similar to that of CSF spaces surrounding the cranial nerves.

FLAIR imaging is more sensitive to subtle T1-shortening caused by, for example, low concentrations of gadolinium or increased protein than T1-weighted (T1W) imaging. 3D FLAIR imaging can provide more detailed anatomical information than 2D FLAIR imaging because 3D sequences offer higher spatial resolution and generate high-quality multiplanar and 3D reconstructions [7,8]. In addition, 3D FLAIR imaging can reduce CSF flow artifacts compared to 2D FLAIR imaging [8]. HT2-FLAIR imaging, a type of 3D FLAIR imaging, is more sensitive to subtle T1-shortening than conventional 3D FLAIR imaging [2-5]. This technique has been applied to various regions such as CSF spaces [2,3], the inner ear [4], and CSF leaks [5,6]. To the best of our knowledge, this is the first study to identify the enhancement of the olfactory recess using HT2-FLAIR imaging.

Understanding CSF and solute dynamics can contribute to the diagnosis and treatment of various diseases. Evaluation of contrast enhancement in intracranial structures has the potential to provide insight into the dynamics. Gadolinium can be deposited in the brain after intravenous administration. Although the precise mechanism is not fully understood, a recent study proposed a gadolinium inflow route via the CSF as well as direct inflow from the blood [9]. In addition, several studies using HT2-FLAIR imaging proposed circumventricular organs [2,10] and superficial veins [11] as candidates for leakage sites into the CSF. Thus, gadolinium may move from the blood into the olfactory recess in our case. Subsequently, the following question arises: How does gadolinium accumulate within the olfactory recess after moving into the CSF from the blood? One possible explanation is that the accumulation of gadolinium may be caused by reduced CSF flow in the olfactory recess, which may also facilitate the visualization of contrast enhancement because 3D FLAIR is sensitive not only to low concentrations of gadolinium but also to slow flow [2,5]. Previous studies have revealed that substances are injected into the CSF drain along cranial nerves [12]. Therefore, gadolinium within the olfactory recess may drain along the olfactory nerves through the cribriform plate.

In this study, contrast enhancement was observed in the CSF spaces surrounding the cranial nerves on postcontrast imaging. This finding contrasts with the results of a previous study by Naganawa et al. [3], which showed that various CSF spaces, such as the subarachnoid space surrounding the optic nerve and Meckel’s cave, did not statistically significantly differ between precontrast and 0.5-h postcontrast HT2-FLAIR imaging. This discrepancy may be attributed to differences in sample size between the two studies. Since the previous study had a small sample size of only six participants, a larger sample size may have yielded a significant difference.

In our study, contrast enhancement was observed on both the postcontrast and 4-h delayed postcontrast imaging in the CSF spaces surrounding the cranial nerves, including the olfactory recess. Notably, contrast enhancement was statistically significantly greater on 4-hour postcontrast imaging compared to that on postcontrast imaging. On the other hand, the olfactory sulcus, another CSF space, had a slight contrast enhancement, although there was no statistically significant difference in relative enhancement between postcontrast and 4-h delayed postcontrast imaging. One possible reason for these different enhancement patterns may be associated with differences in CSF flow. As mentioned earlier, 3D FLAIR is sensitive to low concentrations of gadolinium and slow flow [2-5]. The olfactory sulcus is larger than the CSF spaces surrounding the cranial nerves, which may be related to less flow stasis. If the olfactory sulcus contains high-flow CSF, HT2-FLAIR imaging may have reduced sensitivity to gadolinium detection in the CSF.

Many animal studies have provided evidence that the nasal lymphatic outflow is the major route for CSF efflux via the cribriform plate to the lymphatic system of the nasal mucosa. In humans, classically, CSF was believed to egress predominantly through arachnoid granulation into the dural venous sinuses. However, there is no evidence of CSF outflow into the blood under in vivo physiological conditions [1]. Although several different CSF outflow pathways have been proposed in humans [13], there is limited knowledge of these pathways, unlike in animals. Moreover, further elucidation is required to understand the relative contribution of different outflow pathways to overall CSF egress. Several studies have investigated CSF outflow to the nasal mucosa in human cadavers [14,15] and in vivo in humans [16,17]. These postmortem studies suggest the existence of nasal CSF outflow pathways in humans. However, cadaveric models may not fully reflect the in vivo human physiology, and it is crucial to determine the presence of nasal CSF outflow pathways through in vivo human studies. In an in vivo MR study, gadobutrol was observed in CSF near the cribriform plate on T1W imaging following intrathecal administration in all patients [16]. In nearly half of the patients, gadobutrol was also identified as minute tubular structures inferior to the cribriform plate, probably representative of gadolinium along perineural spaces. However, the nasal mucosa did not show any contrast enhancement. The results of an in vivo human study using dynamic positron emission tomography are in contrast with those of the MR study. 18F-THK5117, a radiotracer that freely crosses the blood-brain barrier, egressed CSF into the superior turbinates after intravenous administration [17]. Moreover, the Alzheimer's disease group showed reduced CSF clearance at both the lateral ventricle and superior turbinates compared with the control group.

This disparity between these two in vivo human studies may have been caused by the types of tracers, sensitivities of the tracers, spatial resolutions, or scan times. In the in vivo MR study, the nasal mucosa showed no contrast enhancement on T1W imaging after intrathecal gadolinium administration. However, due to the lower sensitivity of T1W imaging to subtle T1-shortening, this technique could not detect low concentrations of gadolinium in the nasal mucosa. Alternatively, FLAIR imaging may allow better visualization of gadolinium efflux into the nasal mucosa. In the current study, the nasal mucosa was also enhanced on post-contrast and 4-h delayed post-contrast HT2-FLAIR imaging in all cases (data not shown). This phenomenon may be caused mainly by gadolinium accumulation directly from the blood, although the contribution of nasal lymphatic outflow via the cribriform plate cannot be completely excluded.

Our results may contribute to elucidating the fundamental mechanisms and clinical implications of CSF dynamics and related disorders. Our study revealed that contrast enhancement of the olfactory recess increased over time using HT2-FLAIR imaging. If CSF and gadolinium within the olfactory recess drain along the olfactory nerves through the cribriform plate, this enhancement pattern could be altered in patients with CSF-related neurological disorders (e.g., hydrocephalus) or neurodegenerative diseases (e.g., Alzheimer’s disease). Similarly, since the other cranial nerves are potential candidates for CSF outflow pathways [13], the above-mentioned patients may demonstrate different enhancement patterns of the CSF spaces surrounding these nerves from those shown in our study. The altered enhancement can potentially highlight the diagnostic, prognostic, and therapeutic significance of these conditions.

Limitations

Several limitations in our study should be addressed. First, the relatively small sample size and the patient selection of inclusion and exclusion criteria may have limited the generalizability of our findings. Second, the manual placement of ROIs to measure the SI might be susceptible to operator bias. The measured structures were relatively small and had complex shapes, and there were individual anatomical variations among participants. These factors could potentially introduce inconsistencies in the ROI placement and subsequent SI measurements. Furthermore, all image analyses were performed once by a single observer. Although a consistent method was applied throughout the study, the absence of an evaluation of inter-rater and intra-rater reliability may limit the reproducibility of the findings. Future studies using these reliability methods are warranted to further strengthen the robustness of the measurements. Additionally, data were collected at a few time points. More frequent and extended time point sampling in future studies would provide deeper insight into the temporal dynamics of contrast enhancement and better characterize the physiological processes involved. Finally, while the study provides novel findings, some potential confounding factors, such as physiological variations or underlying pathologies, were not fully controlled or explored in detail. Addressing these variables in future studies would improve the interpretability and external validity of the results.

## Conclusions

Gadolinium was identified in the olfactory recess following intravenous administration using HT2-FLAIR imaging. Our findings may provide novel insights into the dynamics of CSF and solutes, potentially influencing future diagnostic approaches. Further research is needed to explore the clinical implications of olfactory recess enhancement in diagnosing CSF-related disorders and refining imaging protocols.

## References

[1] Mehta NH, Sherbansky J, Kamer AR (2021). The brain-nose interface: a potential cerebrospinal fluid clearance site in humans. Front Physiol.

[2] Osawa I, Kozawa E, Yamamoto Y (2022). Contrast enhancement of the normal infundibular recess using heavily T2-weighted 3D FLAIR. Magn Reson Med Sci.

[3] Naganawa S, Suzuki K, Yamazaki M, Sakurai Y (2014). Serial scans in healthy volunteers following intravenous administration of gadoteridol: time course of contrast enhancement in various cranial fluid spaces. Magn Reson Med Sci.

[4] Osawa I, Kozawa E, Tanaka S (2021). Signal and morphological changes in the endolymph of patients with vestibular schwannoma on non-contrast 3D FLAIR at 3 Tesla. BMC Med Imaging.

[5] Osawa I, Kozawa E, Mitsufuji T, Yamamoto T, Araki N, Inoue K, Niitsu M (2021). Intravenous enhanced 3D FLAIR imaging to identify CSF leaks in spontaneous intracranial hypotension: comparison with MR myelography. Eur J Radiol Open.

[6] Osawa I (2023). Diagnostic imaging of cerebrospinal fluid hypovolemia: the evolution of magnetic resonance imaging and image interpretation [Japanese]. Auton Nerv Syst.

[7] Osawa I, Mitsufuji T, Nagawa K, Hara Y, Yamamoto T, Araki N, Kozawa E (2024). Comparing 2-dimensional versus 3-dimensional MR myelography for cerebrospinal fluid leak detection. Eur J Radiol Open.

[8] Osawa I, Nagawa K, Hara Y, Shimizu H, Tanaka S, Kozawa E (2023). Utility of contrast-enhanced 3D STIR FLAIR imaging for evaluating pituitary adenomas at 3 Tesla. Eur J Radiol Open.

[9] Rasschaert M, Weller RO, Schroeder JA, Brochhausen C, Idée JM (2020). Retention of gadolinium in brain parenchyma: pathways for speciation, access, and distribution. A critical review. J Magn Reson Imaging.

[10] Naganawa S, Taoka T, Kawai H, Yamazaki M, Suzuki K (2018). Appearance of the organum vasculosum of the lamina terminalis on contrast-enhanced MR imaging. Magn Reson Med Sci.

[11] Ohashi T, Naganawa S, Ogawa E, Katagiri T, Kuno K (2019). Signal intensity of the cerebrospinal fluid after intravenous administration of gadolinium-based contrast agents: strong contrast enhancement around the vein of Labbe. Magn Reson Med Sci.

[12] Lochhead JJ, Davis TP (2019). Perivascular and perineural pathways involved in brain delivery and distribution of drugs after intranasal administration. Pharmaceutics.

[13] Proulx ST (2021). Cerebrospinal fluid outflow: a review of the historical and contemporary evidence for arachnoid villi, perineural routes, and dural lymphatics. Cell Mol Life Sci.

[14] Löwhagen P, Johansson BB, Nordborg C (1994). The nasal route of cerebrospinal fluid drainage in man. A light-microscope study. Neuropathol Appl Neurobiol.

[15] Johnston M, Zakharov A, Papaiconomou C, Salmasi G, Armstrong D (2004). Evidence of connections between cerebrospinal fluid and nasal lymphatic vessels in humans, non-human primates and other mammalian species. Cerebrospinal Fluid Res.

[16] Melin E, Eide PK, Ringstad G (2020). In vivo assessment of cerebrospinal fluid efflux to nasal mucosa in humans. Sci Rep.

[17] de Leon MJ, Li Y, Okamura N (2017). Cerebrospinal fluid clearance in Alzheimer disease measured with dynamic PET. J Nucl Med.

